# STOX1 Overexpression in Choriocarcinoma Cells Mimics Transcriptional Alterations Observed in Preeclamptic Placentas

**DOI:** 10.1371/journal.pone.0003905

**Published:** 2008-12-11

**Authors:** Virginie Rigourd, Caroline Chauvet, Sonia T. Chelbi, Régis Rebourcet, Françoise Mondon, Franck Letourneur, Thérèse-Marie Mignot, Sandrine Barbaux, Daniel Vaiman

**Affiliations:** 1 Equipe 21, Institut Cochin, Université Paris Descartes, CNRS (UMR 8104), Paris, France; 2 Inserm, U567, Paris, France; 3 Plate-forme Génomique, Institut Cochin, Université Paris Descartes, CNRS (UMR 8104), Paris, France; 4 Inserm UMR-S747, Université Paris Descartes, Centre Universitaire des Saints Pères 45, Paris, France; University of Calgary, Canada

## Abstract

**Background:**

Mutations in STOX1 were proposed to be causal for predisposing to preeclampsia, a hypertensive disorder originating from placental defects, affecting up to 10% of human pregnancies. However, after the first study published in 2005 three other groups have dismissed the polymorphism described in the first paper as a causal mutation.

**Methodology and Principal Findings:**

In the present study, we have produced a choriocarcinoma cell line overexpressing STOX1. This overexpression results in transcriptional modification of 12.5% of the genes, some of them being direct targets as shown by chromatin immunoprecipitation. STOX1 overexpression correlates strongly and specifically with transcriptomic alterations in preeclamptic placentas (r = 0.30, p = 9.10^−7^). Numerous known key modulators of preeclampsia (such as Endoglin, Syncytin, human chorionic gonadotrophin -hCG-, and Glial Cell Missing Homolog -GCM1-) were modified in these transformed choriocarcinoma cells.

**Conclusions:**

Our results contribute to reconcile contradictory data concerning the involvement of STOX1 in preeclampsia. In addition, they strongly suggest that anomalies in *STOX1* expression are associated with the onset of preeclampsia, thus indicating that this gene should be the target of future studies. Our cellular model could constitute an invaluable resource for studying specific aspects of this human disease.

## Introduction

Mutations in *STOX1* were proposed to be causal for predisposing to preeclampsia [1], a hypertensive disorder originating from placental defects affecting up to 10% of human pregnancies [2]. However, three recent studies have shown that the polymorphism described in the first paper as a causal mutation is widely shared by non-preeclamptic women from various populations [3]–[5]. One of the specific features of *STOX1*, as depicted in the primary study, was its allele-specific expression from maternal alleles, since the authors showed that *STOX1* mRNA was absent in hydatiform moles, which are exclusively of paternal origin [1]. This discovery is in concordance with the idea that imprinted genes may be particularly involved in preeclampsia [6]. However, this observation was challenged by the recent results of Iglesias-Plata and co-workers, which showed by the analysis of SNPs present in *STOX1* ORF, that *STOX1* is rather bi-allelically expressed in the placenta, as well as in other tissues [4]. In the present study, we show in a cellular model that STOX1 overexpression reproduces the transcriptional effects of preeclampsia in the human placenta.

## Results

We stably transfected *STOX1* ORF under the control of the CMV promoter in the JEG-3 choriocarcinoma cell line. The choice of this cell line was dictated by two considerations; first of all, a transformed line is a tool that can be kept and used by several teams; second, JEG-3 cells are considered as a good model for trophoblasts, and have been used in numerous studies on this topic. Interestingly, JEG-3 cultures release human chorionic gonadotrophin, human placental lactogen, progesterone, and are able to transform steroid precursors into estrone and estradiol. They have been profusely used to investigate molecular aspects of trophoblast function.

**Figure 1:**
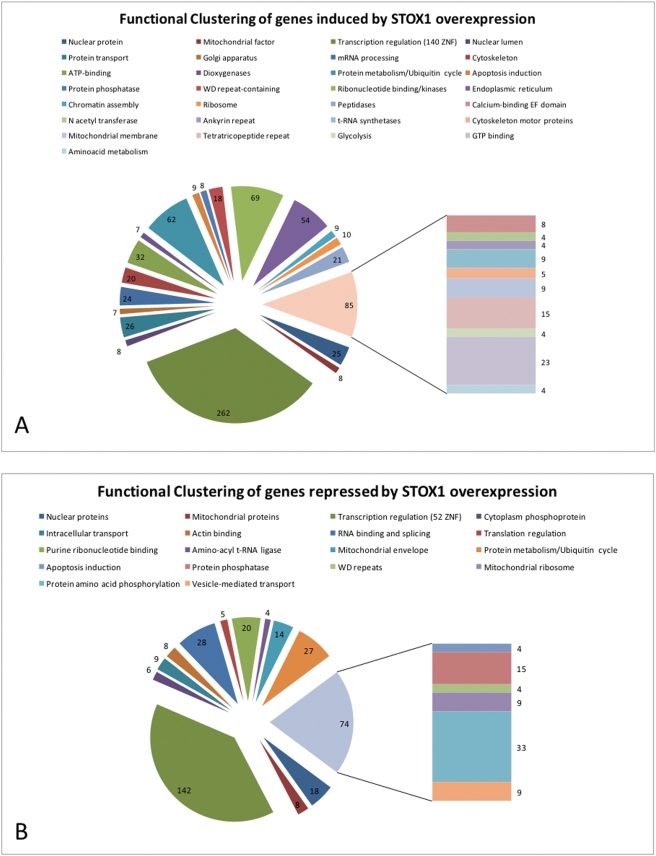
Fonctional clustering of genes modified by STOX1 overexpression (a total of 1840 induced genes and 1018 repressed genes) were distributed in clusters using the DAVID website [7]. Interestingly, the major cluster was in both cases composed of transcription factors, among which a majority of Zinc Finger Proteins was found. Only the significant clusters (according to the threshold defined in Material and Methods are represented), and are classified by decreasing significance (clockwise).

Nine stable clones expressing STOX1 were obtained, with overexpressions ranging from 2 to 30 fold, as shown by qRT-PCR. Total RNA was prepared from the AA6 cell line (overexpressing STOX1 30 times) and the BD3 cell line (stably transfected with the empty pCMX vector). mRNAs were used as templates to label cDNA that was hybridized to Affymetrix arrays (u133 2.0) encompassing 53,000 human transcripts. The results have been deposited as a GEO dataset, with the accession number GSE13475. Among the 53,000 transcripts, 22,980 were expressed at a sufficient level to allow their detection and analysis. We found that more than 2800 transcripts (∼12.5%) were significantly modified by *STOX1* over expression (1840 induced –figure 1A- and 1018 repressed –figure 1B- at least two-fold).

We used the DAVID software on the groups of induced and repressed genes (http://david.abcc.ncifcrf.gov/home.jsp) [7] to detect Gene Ontology groups. We could show a significant excess of transcription factors among the genes that were successfully grouped in clusters (415 repressed (39.9%), 848 induced (46.1%), Figure 1). Indeed, inside these groups, 168 (30%) and 255 (40.4%) genes were related to transcription regulation among repressed and induced genes, respectively. Among the modified transcription factors, Zinc Finger-containing genes were very abundant (56 and 131, respectively). This proportion was highly significant compared to other families of transcription factors (Table 1), and associated with an excess of induced transcripts. Reciprocally, we observed that a high number of Homeobox-containing genes were down-regulated. Other analyzed families were not significantly modified compared with the rest of the transcripts.

**Table 1:** Transcriptional factors modified by STOX1 overexpression.

Category of transcription factor	Up-regulated (>2-fold induction)	Down-regulated (<2-fold decrease)	Unchanged	Chi2 test
ZNF-related	127	26	774	**6.9 10^−10^**
FKHD-related	4	4	30	0.064
Homeobox-containing	4	8	63	**0.009**
SOX-related	0	0	12	186
TATA-related	1	3	40	0.122
All transcriptome	1840	1018	20 122	

**Figure 2:**
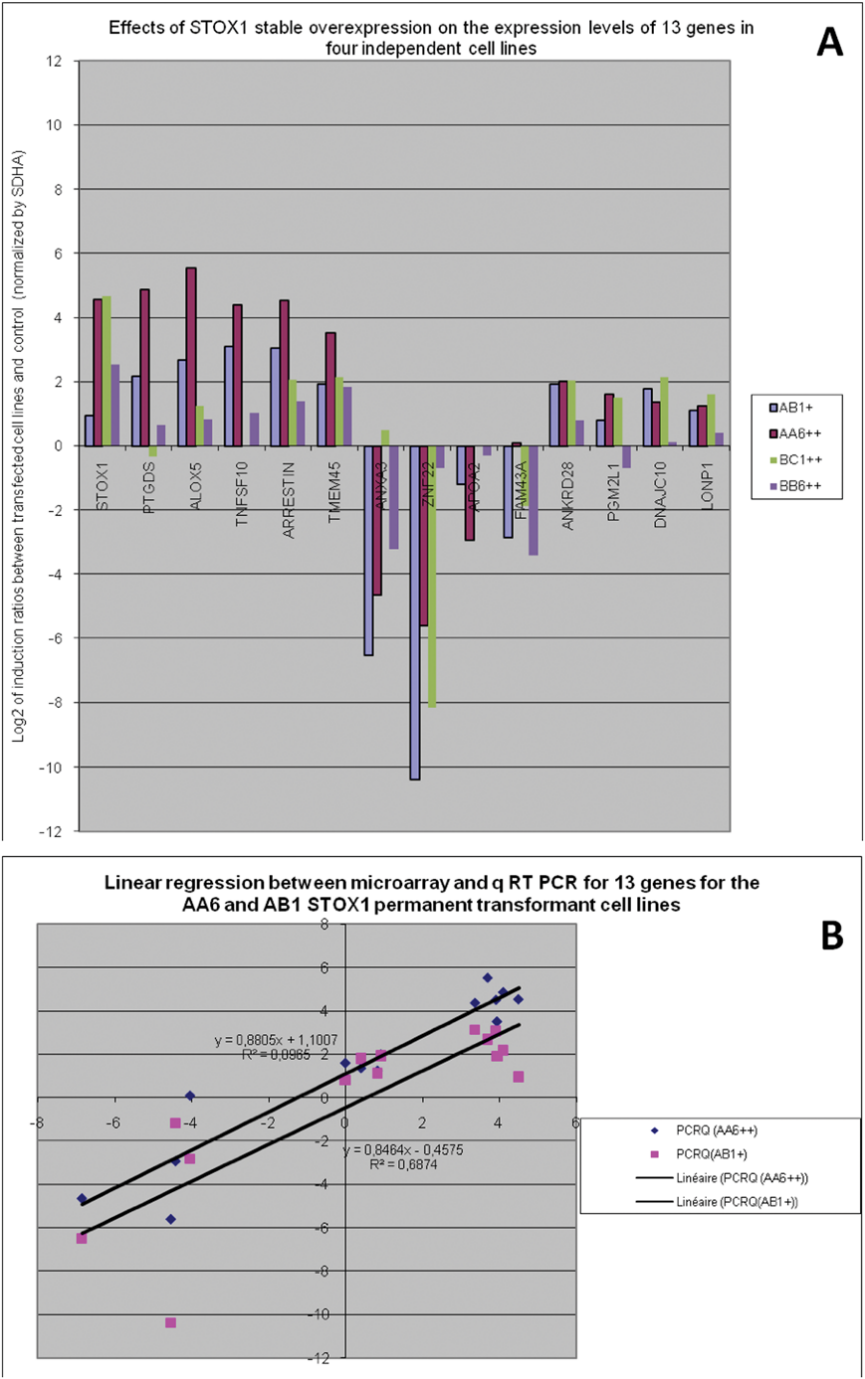
(A) Comparison of four cell lines overexpressing STOX1 for a series of 13 genes known to be modified by the microarray experiment, by quantitative RT-PCR. Five genes were overexpressed (*PTGDS*, *ALOX5*, *TNFSF10*, *βARRESTIN*, *TMEM45a*), four down-regulated (*ANXA3*, *ZNF22*, *APOA2*, *FAM43A*) and four mildly induced or unmodified (*ANKRD28*, *PGM2L1*, *DNAJC10*, *LONP1*). The diagram shows a fairly good reproducibility of the gene alterations whatever the cell line overexpressing STOX1. The expression level of STOX1 is represented in the first series of histograms. (B) Comparison of the expression levels of the microarray and of the qRT-PCR results for the same series of 13 genes. For AA6, the cell line from which the cDNA hybridized on the microarrays was obtained, the correlation is excellent (R = 0.944). For the second line AB1, overexpressing STOX1 at a milder level, the correlation is also very good (R = 0.829). In this case, the overall expression is nevertheless lower as shown by the linear regression. This suggests that at least in some cases, the deregulations induced by STOX1 overexpression are somehow proportional to this overexpression.

In addition, we used qRT-PCR on some of the stable transformants expressing *STOX1* at different levels, and obtained an external validation of the microarray results (Figure 2). We analyzed a subset of five induced, four repressed and four unmodified genes. The correlation between the Log_2_ (induction) in the array compared with the Log_2_ (induction) in the qPCR experiment was 0.944 (Figure 2B). Despite some individual variations between stable transformants, STOX1 overexpression induced similar gene alterations (Figure 2A). This indicates that the effects noticed in the two cell lines used for the microarray are directly caused by STOX1 and not by possible general deregulations induced by the transfection and selection procedure.

**Figure 3:**
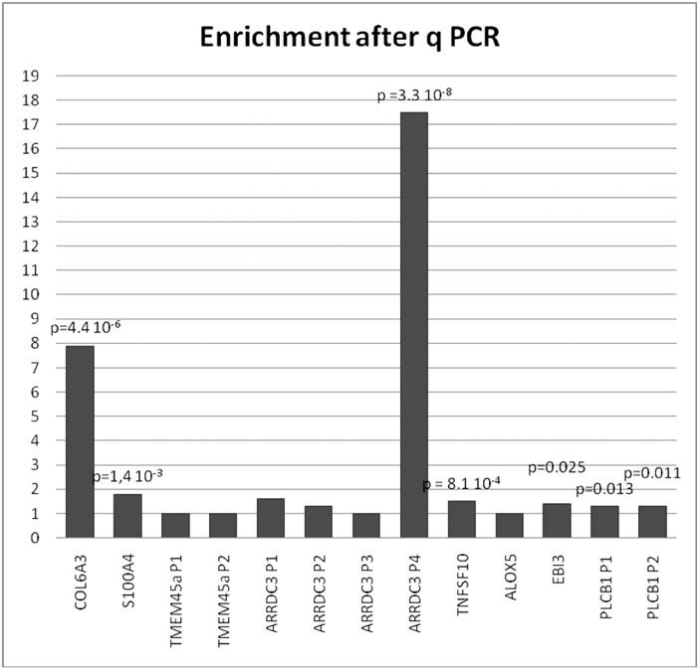
Thirteen promoters encompassing putative binding sites for Forkhead containing transcription factors were quantified by qPCR after Chromatin Immunoprecipitation, using a novel anti-STOX1 polyclonal antibody. For several genes alternative putative promoters were identified by Genomatix, and only some of the promoters appeared enriched. This is clearly the case for *ARRDC3*, for which promoter P4 was considerably and exclusively enriched (17.5 fold compared to control). Some other targets were only mildly enriched such as the *TNFSF10* promoter. The results are the mean of four independent ChIP experiments on which three qPCR assays have been performed.

Next, we analyzed systematically the promoter composition of transcription factors whose expression is affected by STOX1 using Genomatix (www.genomatix.de). We contrasted the group formed by induced (55 promoters) and repressed (39 promoters) transcription factors with non-modified genes (49 promoters) by a Student t-test (Supplemental Table S1). For binding sites present on average more than once in each promoter, the forkhead binding site (V$FKHD) was the most significant (p = 0.0044) after V$BRNF, a binding site for POU transcription factors (p = 0.0022). This observation is consistent with the idea that STOX1 belongs to the enlarged FOX family. We then performed ChIP experiments (four independent experiments) followed by qPCR using primers from the promoters of genes strongly induced by *STOX1* overexpression and enriched in FKHD binding sites (Figure 3). Thirteen different promoters corresponding to the 8 genes *COL6A3*, *S100A4*, *TMEM45a*, *ARRDC3*, *TNFSF10*, *ALOX5*, *EBI3* and *PLCB1* (induced 28-, 19-, 15-, 15-, 9-, 13-, 12-, and 11 fold, respectively), were recovered using the Genomatix Gene2Promoter function. For several genes, multiple putative promoters were identified by the program (up to 4 for *ARRDC3*) and primers surrounding the putative forkhead binding sites were designed. Among the thirteen putative promoters, seven were significantly enriched after chromatin IP and corresponded to 6 genes out of 8. In the case of *ARRDC3*, only one promoter was significantly enriched by immunoprecipitation. This approach confirmed that *STOX1* binds the promoter of these genes as a transcription factor involved in their regulation.

**Figure 4:**
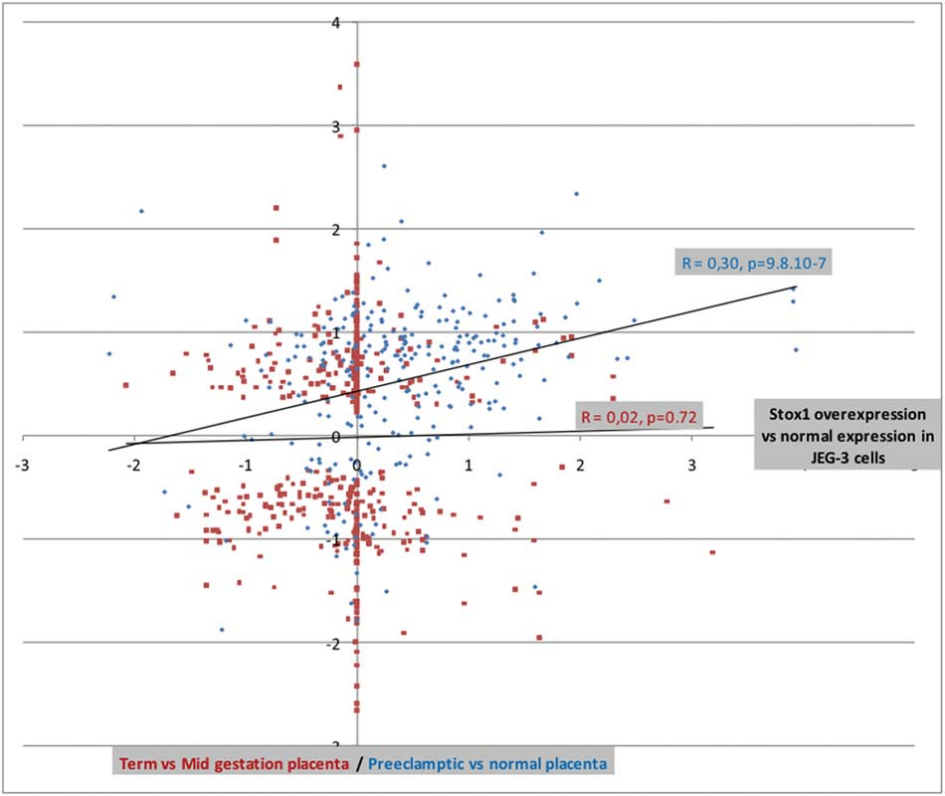
Linear regression analysis comparing the induction/repression ratio of the genes from the present study (comparing JEG-3 cells overexpressing STOX1 with mock-transfected JEG-3 cells) with induction ratio of two other transcriptomic studies on the placenta. The first external study, performed by Nishizawa and coworkers [8] compared 4 normal and 10 preeclamptic placentae. The 500 most modified genes from this article were obtained, and 265 were detectable among the genes identified here. The induction/repression ratio of the 265 genes are plotted (horizontal axis, blue diamonds) against the same genes of the present work (vertical axis). In this case, the correlation was highly significant between the two sets of induction ratio (r = 0.30, p<0.000001). As a control, another study on placental transcriptomics was used, which compares mid gestation and term placentas [11]. Again, the 500 most modified genes were selected and 311 were detectable among the genes identified in the present study. Their induction/repression ratio are represented along the horizontal axis as red squares. In this case, the correlation was very close to 0 (r = 0.02, p = 0.72). These results demonstrate that STOX1 overexpression in choriocarcinoma reproduces with fidelity several aspects of the preeclamptic disease despite the much higher complexity of the placenta.

We then attempted to compare our microarray results with published transcriptomic data comparing normal and preeclamptic placentas. Among these few studies, only one to the best of our knowledge was deposited as accessible GEO Profiles [8]. We extracted the 500 genes that were the most different between normal and preeclamptic placentas from this study. Among those, 265 (207 induced and 58 repressed in preeclampsia) were expressed at a sufficient level to be detected in our microarray experiment (Supplemental Table S2a). These values enabled us to show very significant differences between the expected proportions of induced or repressed genes if the distribution was random (Supplemental Table S3). The data were also analyzed by a linear regression approach (Figure 4). To achieve this, the induction ratio of the 265 genes found to be transcriptionnally modified in the preeclampsia study of Nishizawa and co-workers [8], were plotted against the ratio of induction/down-regulation of the same genes in the JEG-3 cells overexpressing *STOX1*. Strikingly, the correlation applied between genes whose expression is affected by STOX1 and preeclampsia-modified genes was highly significant (r = 0.295, p = 9.8 10^−7^, Figure 4). Since this correlation was made between induction/repression ratios, it is corrected for confounding effects, such as gene expression levels for instance. Very similar results were obtained when comparing STOX1 effects with transcriptional deregulation observed in older transcriptomic studies dealing with syncytialisation and the links between hypoxia and placental diseases [9], [10].

As a control of the specificity of STOX1-induced transcriptome alterations, our data were compared with a study where mid-gestation versus normal term placentas were analyzed by microarray analysis [11]. We also extracted the 500 most modified genes from this study and 311 were common with the ones of our own experiment (Supplemental table S2b). However, in this case, no correlation could be found between the subsets of transcriptionnally modified genes (r = 0.02 p = 0.72, Figure 4). These observations strongly suggest that STOX1 overexpression in JEG-3 cells reliably reproduces modifications induced by preeclampsia in the placenta.

## Discussion

Our results provide new data suggesting that STOX-1 could be involved in the processes leading to preeclampsia. Interestingly, among the modified genes, we found i) hCG (induced 2.25 fold for CGA and 3.23 fold for CGB, the hCG-specific polypeptide), one of the well-known overexpressed genes in preeclamptic placentas, ii) endoglin (induced 2.23 fold), which is the earliest serum marker observed as induced by preeclampsia in patients [12], iii) ERVW1 (syncitin, induced 2.03-fold), an endo-retrovirus known to be crucial for the formation of the syncytiotrophoblast [13], as well as iv) GCM1 (decreased 3.4-fold), known as the most specific transcription factor for trophoblast cells [14], and specifically down-regulated in preeclamptic placentas [15]. In addition, overexpression of STOX1 stimulates EBI3 (×12.0-fold), an interleukin–12 p40 expressed at very high level by syncytiotrophoblasts and extra villous trophoblasts throughout human pregnancy, and involved in implantation. It is presented by HLA-G, suggesting that it is an immunomodulator, possibly involved in NK cell regulation able to modulate proliferation and migration processes [16; 17]

Similar to other studies [3]–[5] challenging the role of STOX1 in preeclampsia, we sequenced 20 patients and 20 controls but could not find any association with the Y153H polymorphism described in the first study [1], neither find any transcriptional effects differentiating normal and pathological placentas (data not shown). We hypothesize that the reported polymorphism is in linkage disequilibrium with the true causal mutation. It is interesting to notice that STOX1 in mice and humans contains a very long first intron (∼50 kb), which has not been sequenced, and could contain elements regulating the mRNA splicing, shown to be important for STOX1 sub-cellular localization [1]. Since the transcription level did not vary between normal and pathological placentas, our results suggest that STOX1 is involved in very early events of placental development, which could not be addressed by analyzing term or near-term placentas. Another problem reported by Iglesias-Plata is the non-imprinted status of STOX1 [4]. In fact, a close examination of their sequencing data suggests that there could be a quantitative difference in allelic expression in fetal and placental tissues. This could be explained by cell-type heterogeneity of its imprinted status in the placenta. Although they also show that the first CpG island located in the first exon is unmethylated, other distant regulatory regions could be altered as reported for the IGF2/H19 system.

Caution is warranted when extrapolating results obtained in a cellular model to the general trophoblast population. However, JEG-3 cells constitute an interesting and reliable model of trophoblast function, as suggested by ancient and recent studies [18]. The results of the present study, substantiate this idea, since the main result is a strong and significant correlation between a specific alteration of the JEG-3 transcriptome and preeclampsia-induced transcriptional modifications in the placenta. Clearly, while this correlation is highly significant, the rest of the variability could be explained by the action of other pathways involved in preeclampsia, as well as by modifications of other cell types present in the placental villi. In this context in vitro transformation of cytotrophoblasts into syncytiotrophoblast, as previously described [19]–[22] seem to show a specific and exclusive expression of *STOX1* in the former (Rigourd et al, in preparation). It would mean that the syncytialisation process abruptly inactivates the expression of STOX1, the underlying mechanisms remaining elusive. Since hypoxia, or more generally brutal variation in oxygen pressure, are considered as major actors of preeclampsia pathogenesis [23; 10; 24], it was interesting to match the microarray result with HIF1α induction. However, HIF1α was not at all modified at the transcriptional level by *STOX1* overexpression. This does not disqualify an effect on the stabilization of the HIF1α protein, which could then participate in the gene induction effects. Also, using cDNA from placental explants put in hypoxia, we could not see any induction of *STOX1* expression (data not shown). Hypoxia-induced genes were nevertheless found elevated in preeclampsia [10], and were significantly correlated with gene-induction in the JEG-3 cells overexpressing *STOX1* (present study). Therefore, the link between hypoxia and *STOX1* exists, but probably in a subtle form. In conclusion, our study indicates that STOX1 deserves further investigation for its role in the aetiology of preeclampsia.

## Materials and Methods

### Patients and ethics

All the placentas from the patients were collected from four Parisian maternities (Cochin, St Antoine, Institut de Puériculture and St Vincent de Paul). This study was approved by the Ethics Committee of Paris Cochin (France), CCPPRB (Comité Consultatif de Protection des Personnes dans la Recherche Biomédicale). All patients have given their written consent for the use of their placenta. Placentas were obtained from caesarean section outside labor from healthy mothers.

### Placental villi and cytotrophoblast purification

Biopsy samples were rapidly collected at six to ten various locations from each placenta between the decidual and chorionic plates. Villous tissue was dissected free of fetal membranes, vessels and tissue from maternal origin, rinsed and minced in Ca^2+^, Mg^2+^ free Hank's Balanced Salt Solution (HBSS). Cytotrophoblast cells were prepared using standard procedures of negative selection mastered in the laboratory [19]–[22].

### Plasmid constructions

The STOX1A construct was a generous gift of Dr C.B. Oudejans (VU University Medical Center, De Boelelaan 1117, 1081 HV, Amsterdam, The Netherlands). The ORF was isolated and subcloned in the pCMX expression vector and resequenced. Three mutations were found (A→C in 1658 (GAA = Glu→GCA = Ala), a deleted T in 2948 (which creates an early stop codon), and the initiation codon (ACG (THR) instead of ATG (MET)) and corrected by site-directed mutagenesis. Hence we obtained a pCMX vector containing the coding region of STOX1-A.

### Cell culture and stable transfection

JEG-3 choriocarcinoma cells were seeded in DMEM medium (Gibco) supplemented with 10% FBS and 1% penicillin/streptomycin at a concentration of 1.0×10^6^ cells per T25 culture flask. At the time of transfection cells were at 60% confluence. Cells were transfected using Lipofectamin® Reagent (Invitrogen), according to the manufacturer recommendations with 4 µg of pCMX-STOX1-A or 4 µg empty pCMX together with 0.4 µg of PGK neo (the PGK-neo expression vector was a kind gift of Dr Djian [25]) per T 25 cm^2^ culture dish with Opti-MEM® I Reduced Serum Medium. This plasmid ratio is known to ensure a co-transfection by both plasmids when the cells become resistant. Cells were passaged at 1∶10 dilution into selective medium 72 hours post transfection. Selection was continuously applied using Geneticin (G-418) (Invitrogen) at 500 µg/ml concentration for approximately 3 weeks. Resistant clones (9 cell lines transfected with pCMX-STOX1-A and 3 cell lines transfected with empty pCMX) were grown individually in continual selection and used for further analysis or frozen in DMSO.

### RNA Extraction and cDNA synthesis

Total RNA was extracted from 12 differents clones using Trizol Reagent (Invitrogen) in accordance with the manufacter's instruction. cDNA synthesis was performed using Superscript cDNA synthesis kit (Invitrogen).

### Quantitative RT-PCR

q RT PCR was performed using a Light Cycler (Roche) and the Invitrogen kit (SYBR®). The primers for STOX1 cDNA amplification were: 5′-TTGGGTGTGAA-3′, 5′-TTGGAGCGTTTGATGAAACA-3′. The clone with the highest STOX-1A overexpression (AA6) and a negative clone (BD3) were selected by RT-PCR with SDHA as reference gene. The primer couples for SDHA were 5′-TACAAGGTGCGGATTGATGA-3′, 5′-GCAACAGAAGAAGCCCA-3′.

### Gene expression array

5 micrograms of cDNA of AA6 and BD3 clones were sent to Affymetrix type IVT3 expression array platform. cDNA and labeling for hybridization scanning and data normalization were performed by the platform, which provided the final data. Standard quality control procedures were performed and validated (available upon request). Data have been sent to a public database upon acceptance of the study for publication (GSE 13475 and summarized as supplemental data, Supplemental Table S4).

### Bioinformatics

Putative transcription factor binding sites were identified by statistical analysis (Student t-test, followed by Bonferronni correction for multiple testing) using the Gene2Promoter option of Genomatix (Genomatix.de). Functional clustering was performed using the DAVID database. The groups are clusterized according to common keywords, enabling to define a p-value of the occurrence of the keyword in a specific group of genes inside the set of genes submitted, compared with the total gene content. The calculated enrichment score is the harmonic mean of the inverse of these p-values. Since random groups of genes are also able to generate enrichments, a simulation was performed with random groups of genes which enabled to define a threshold for significance, as previously described [26]. More precisely, twenty groups of randomly selected genes (50 to 3000 genes) were introduced in the “upload” window of the DAVID website and submitted to functional clustering. We could observe that the number of clusters obtained by this approach increased with the number of genes in a linear fashion. Furthermore, the enrichment score calculated for the first cluster obtained (the highest), increased steadily with the number of genes introduced. We could derive a linear equation (Highest enrichment score = 0.002×Number of genes +0.4718, R^2^ = 0.84) able to predict the highest score from random data. This information was used to select gene clusters that present an enrichment score above this value.

### Characterization of an anti-rabbit polyclonal STOX1 antibody

A novel polyclonal antibody was generated using two peptides (EP053592: AcNH-GGR AVF RAF RRA NAR C-CONH2, EP053593 H2N-CDL KPS QTG PKE KPF Q-CONH2) injected in rabbits by Eurogentec (Belgium). This antibody was validated by Western blot and by immunohistochemistry staining. The specificity was assessed using the two free peptides as competitors. Specific bands corresponding to three STOX1 isoforms were detected. *In vitro* synthesis and labeling experiments using the three main STOX1 (A, B, C) isoform-coding plasmids as templates indicated that the bands detected by the antibody in placenta and JEG-3 cell extracts correspond to these three isoforms.

**Chromatin Immunoprecipitation** adapted from [27] JEG-3 cells were seeded at a concentration of 1.0×10^6^ cells per T25 culture flask. The initial step is the crosslinking of the live cells with formaldehyde. Cells were then lysed (Buffer containing HEPES, NaCl, EDTA, Triton, EGTA, SDS and Protease inhibitor cocktail) and released chromatine fragments were sheared by sonication to generate DNA fragments of ∼200 bp. Specific immunoprecipitated factors together with crosslinked DNA were immunoprecipitated at +4°C after an 1 hour incubation with pre-immune serum and a preclearing step with a 50% salmon sperm/BSA/ProteinA beads slurry. Anti-rabbit polyclonal STOX1 antibody at 1/100 concentration was used overnight for immunoprecipitation. Finally, protein-DNA crosslink in the immunoprecipitated (IP) material was reversed and the DNA fragments purified. If the STOX1 protein was associated with a specific genomic region in vivo, DNA fragment from this region should be enriched in IP compared to the other portion of the genome.

### QPCR validation

The presence and quantification of relevant genomic region in the IP is determined by qPCR in which the immunoprecipitated material was used as a template for simultaneous amplification with gene specific primers and from a reference region (micro satellite DXS 118). The screening of 8 genes (up to 4 putative promoters for *ARRDC3*) affected by STOX1 overexpression and containing FKKD putative binding sites in their promoter was performed. The primers were designed by using Primer-3 software (http://frodo.wi.mit.edu/cdi-bin/primer3/primer3-www.cgi. For qPCRs, we used the Absolute QPCR SYBR® green mix (ABgene) and the Roche Light-Cycler PCR apparatus. Results obtained with the precipitated DNA were compared to those from input DNA samples.

## Supporting Information

Table S1 Promoter analysis. Comparison of the promoter content in Transcription factor binding sites betwwen STOX1-modified and STOX-1 unmodified genes
(0.21 MB XLS)

Table S2 Table S2A and S2B: Data for correlation analysis transcriptomic data from two experiments (effects of preeclampsia, and effect of gestation time) compared with STOX1 effects
(0.24 MB XLS)

Table S3 Observed versus expected between Stox1 overexpressed genes and 265 genes strongly modified in preeclamptic placentas (According to Nishizawa et al, 2007). Statsitical comparisons (Chi2 tests) for modified categories of genes at three threshold between preeclampsia effects and STOX1 overexpression effects
(0.02 MB XLS)

Table S4 AA6 column corresponds to the cells overexpressing STOX1. BD3 to the control cells transfected with empty pCMX vector
(4.43 MB XLS)
